# On the Preparations of the Indian Hemp, or Gunjah (Cannabis Indica), Their Effects on the Animal System in Health, and Their Utility in the Treatment of Tetanus and Other Convulsive Diseases

**Published:** 1840-07

**Authors:** 


### Art. XIII.

*On the Preparations of the Indian Hemp, or Gunjah* (*Cannabis Indica*), *their Effects on the Animal System in Health, and their Utility in the Treatment of Tetanus and other Convulsive Diseases.* By W. B. O'Shaughnessy, m.d., Professor of Chemistry in the Medical College, Calcutta.—*Calcutta*, 1839. 8vo, pp. 46.

This pamphlet contains a detail of facts of a very important kind, which, we doubt not, will cause a great sensation among the members of the profession throughout the world. We feel it, therefore, to be a duty to give as full an account of its contents as our space will permit. It will appear clearly from what we shall state that Dr. O'Shaughnessy has the merit of having added to *our* materia medica a drug of great and unequivocal powers, and, probably, a remedy of marked efficacy in diseases hitherto the most unmanageable. For the sake of accuracy, in a matter of so much consequence, we shall avail ourselves, as much as possible, of the author's own words, and endeavour to communicate all the more important facts in a series of extracts from his pamphlet.

"The narcotic effects of hemp are popularly known in the south of Africa, South America, Turkey, Egypt, Asia Minor, India, and the adjacent territories of the Malays, Burmese, and Siamese. In all these countries hemp is used in various forms, by the dissipated and depraved, as the ready agent of a pleasing intoxication. In the popular medicine of these nations we find it extensively employed for a multitude of affections. But in western Europe its use either as a stimulant or as a remedy is equally unknown. .......... Much difference of opinion exists on the question, whether the hemp so abundant in Europe, even in high northern latitudes, is identical in specific characters with the hemp of Asia Minor and India. The extraordinary symptoms produced by the latter depend on a resinous secretion with which it abounds, and which seems totally absent in the European kind. The closest physical resemblance or even identity exists between both plants; difference of climate seems to me more than sufficient to account for the absence of the resinous secretion and consequent want of narcotic power in that indigenous in colder countries.........

"*Chemical Properties.* In certain seasons and in warm countries a resinous juice exudes and concretes on the leaves, slender stems, and flowers. Separate and in masses it constitutes the *churrus* of Nipal and Hindostan, and to this, the type or basis of all the hemp preparations, are the powers of these drugs attributable. The resin of the hemp is very soluble in alcohol and ether; partially soluble in alkaline, insoluble in acid solutions; when pure of a blackish-gray colour; hard at 90°; softens at higher temperatures and fuses readily; soluble in the fixed and in several volatile oils. Its odour is fragrant and narcotic; taste slightly warm, bitterish, and acrid. The dried hemp plant which has flowered, and *from which the resin has not been removed,* is called *gunjah.* It yields to alcohol twenty per 100 of resinous extract, composed of the resin (*churrus*) and green colouring matter (*chlorophylle*). Distilled with a large quantity of water, traces of essential oil pass over, and the distilled liquor has the powerful narcotic odour of the plant. The *gunjah* is sold for smoking chiefly..........

"*Popular Uses.* The preparations of hemp are used for the purpose of intoxication as follows: *Sidhee*, *Subjee*, and *Bang* (synonymous) are used with water as a drink, which is thus prepared. About three tola weight, 540 troy grains, are well washed with cold water, then rubbed to powder, mixed with black pepper, cucumber and melon seeds, sugar, half a pint of milk, and an equal quantity of water. This is considered sufficient to intoxicate an habituated person. Half the quantity is enough for a novice. From either of these beverages intoxication will ensue in half an hour. Almost invariably the inebriation is of the most cheerful kind, causing the person to sing and dance, to eat food with great relish, and to seek aphrodisiac enjoyments. In persons of a quarrelsome disposition it occasions, as might be expected, an exasperation of their natural tendency. The intoxication lasts about three hours, when sleep supervenes. No nausea or sickness of stomach succeeds, nor are the bowels at all affected; next day there is slight giddiness and vascularity of the eyes, but no other symptom worth recording.

"*Gunjah* is used for smoking alone, one rupee weight, 180 grains, and a little dried tobacco are rubbed together in the palm of the hand with a few drops of water. This suffices for three persons. A little tobacco is placed in the pipe first, then a layer of the prepared *gunjah,* then more tobacco, and the fire above all." (pp. 1-8.)

The remarkable qualities of this drug seem to have been well known to the Arabian and Persian physicians, both ancient and modern; but Dr. O'Shaughnessy could nowhere find any account of its mode of administration as a remedy, of its dose, or of its exact therapeutic effects. The first step, therefore, was to institute an extensive series of experiments with it on animals. These satisfactorily exhibited at once the power of the drug (chiefly in producing intoxication and ultimate insensibility), and its safety.

"In none of these or several other experiments was there the least indication of pain or any degree of convulsive movement observed. It seems needless to dwell on the details of each experiment; suffice it to say that they led to one remarkable result—that while carnivorous animals and fish, dogs, cats, swine, vultures, crows, and adjutants invariably and speedily exhibited the intoxicating influence of the drug, the graminivorous, such as the horse, deer, monkey, goat, sheep, and cow, experienced but trivial effects from any dose we administered. Encouraged by these results no hesitation could be felt as to the perfect safety of giving the resin of hemp an extensive trial in the cases in which its apparent powers promised the greatest degree of utility." (p. 20.)

We shall now give a brief extract of the result of Dr. O'Shaughnessy's experience of this remedy on the human subject; noticing, in the first place, the preparations used by him and their doses.

"The *resinous extract* is prepared by boiling the rich adhesive tops of the dried *gunjah* in spirit (sp. gr. 835), until all the resin is dissolved. The tincture thus obtained is evaporated to dryness in a vessel placed over a pot of boiling water. The extract softens at a gentle heat, and can be made into pills without any addition.

"The *tincture* is prepared by dissolving three grains of the extract in one drachm of proof spirit.

"*Doses, &c.* In *tetanus* a drachm of the tincture every half hour until the paroxysms cease or catalepsy is induced. In *hydrophobia* I would recommend the resin in soft pills, to the extent of ten to twenty grains to be chewed by the patient, and repeated according to the effect. In *cholera* ten drops of the tincture every half hour will be often found to check the vomiting and purging, and bring back warmth to the surface. My experience would lead me to prefer *small* doses of the remedy in order to excite rather than narcotize the patient." (p. 37.)

***Rheumatism.*** The remedy was tried in several cases both acute and chronic, but, as appears to us, without very satisfactory results. In one of the cases the most marked catalepsy was produced, besides the usual intoxicating effects.

"In several [other] cases of acute and chronic rheumatism admitted about this time, half-grain doses of the resin were given, with closely analogous effects: alleviation of pain in most, remarkable increase of appetite in all, unequivocal aphrodisia, and great mental cheerfulness. In no one case did these effects proceed to delirium, nor was there any tendency to quarrelling. The disposition developed was uniform in all, and in none was headach or sickness of stomach a sequel of the excitement." (p. 24.)

*Hydrophobia.* This was an undoubted case of the disease and was full-formed before the remedy was given. The results are certainly most cheering, although death was not averted.

"By his own desire water was brought in a metallic vessel, which he grasped and brought near his lips; never can I forget the indescribable horrors of the paroxysm which ensued. It abated in about three minutes, and morbid thirst still goading the unhappy man, he besought his servant to apply a moistened cloth to his lips. Intelligent and brave, he determinately awaited the contact of the cloth, and for a few seconds, though in appalling agony, permitted some drops to trickle on his tongue, but then ensued a second struggle, which, with a due share of the callousness of my profession, I could not stand by to contemplate. Two grains of hemp resin in a soft pillular mass were ordered every hour; after the third dose he stated that he felt commencing intoxication; he now chatted cheerfully on his case, and displayed great intelligence and experience in the treatment of the very disease with which he was visited. He talked calmly of drinking, but said it was in vain to try, but he could suck an orange; this was brought to him, and he succeeded in swallowing the juice without any difficulty. The hemp was continued till the sixth dose, when he fell asleep, and had some hours' rest. Early the ensuing morning, however, Mr. Siddons, my assistant, was called up to him, and found him in a state of tumultuous agony and excitement. The hemp was again repeated, and again by the third dose the cheering alleviation of the previous day was witnessed. He ate a piece of sugar-cane and again swallowed the juice; he partook freely of some moistened rice, and permitted a purgative enema to be administered. His pulse was nearly natural, the skin natural in every respect. His countenance was happy.

"Four days thus passed away, the doses of hemp being continued. When he fell asleep on waking the paroxysms returned, but were again almost immediately assuaged as at first. Meanwhile purgative enemata were employed, and he partook freely of solid food, and once drank water without the least suffering. But about 3 p. m. of the fifth day he sunk into profound stupor, the breathing slightly stertorous; in this state he continued, and without further struggle death terminated his sufferings at 4 a. m. on the 27th November." (pp. 24-6.)

Dr. O'Shaughnessy's remarks on this interesting case seem well warranted, namely, "that at least one advantage was gained from the use of the remedy—the awful malady was stripped of its horrors; if not less fatal than before, it was reduced to less than the scale of suffering which precedes death from most ordinary diseases." (p. 26.)

*Cholera.* The epidemic in a few cases of which the hemp was tried was mild, and the results are therefore inconclusive; but they are, as Dr. O'Shaughnessy says, "*promising*, and deserve the attention of the practitioner."

*Tetanus.* Several cases of the traumatic kind are recorded, which cannot fail to excite the highest expectations of the power of this remedy in this generally fatal disease.

In the first case, the disease supervened on dysentery from the action of a native moxa. Symptoms of tetanus occurred on the 24th Dec., and were well marked on the 26th, when (the case being considered hopeless) hemp was administered, at first in doses of two grains every third hour, afterwards of three grains every second hour. The usual intoxicating effects were produced, and the spasms were speedily mitigated, and at length finally ceased on the 6th Jan. The dysentery, however, proved fatal on the 23d.

"The *second case* was that of Chunoo Syce, in whom tetanus supervened on the 11th December, after an injury from the kick of a horse. After an ineffectual trial of turpentine and castor oil in large doses, two-grain doses of hemp resin were given on the 26th November. He consumed in all 134 grains of the resin, and left the hospital cured on the 28th December.

"*Third Case.* Huroo, a female æt. 25, admitted to the Native Hospital on 16th December, had tetanus for the three previous days, the sequel of a cut on the left elbow received a fortnight before. Symptoms violent on admission. Turpentine and castor oil given repeatedly without effect; on the 16th and 17th, three grains of hemp resin were given at bed-time. On the morning of the 18th she was found in a state of complete catalepsy, and remained so until evening, when she became sensible, and a tetanic paroxysm recurred. Hemp resumed, and continued in two-grain doses every fourth hour. From this time till the third hour tetanic symptoms returned. She subsequently took a grain twice daily till the 8th of February, when she left the hospital apparently quite well.

"Mr. O'Brien has since used the hemp resin in five cases, of which four were admitted in a perfectly hopeless state. He employed the remedy in *ten-grain doses* dissolved in spirit. The effect he describes as almost immediate relaxation of the muscles and interruption of the convulsive tendency. Of Mr. O'Brien's seven cases, four have recovered.

"In the Police Hospital of Calcutta, the late Dr. Bain has used the remedy in three cases of traumatic tetanus, of these, one has died and two recovered.

"A very remarkable case has recently occurred in the practice of my cousin, Mr. Richard O'Shaughnessy. The patient was a Jew, æt. 30, attacked with tetanus during the progress of a sloughing sore of the scrotum, the sequel of a neglected hydrocele. Three-grain doses were used every second hour, with the effect of inducing intoxication and suspending the symptoms. The patient has recovered perfectly, and now enjoys excellent health. (p. 31.)

We think no unprejudiced reader of Dr. O'Shaughnessy's cases of tetanus can hesitate to concur with him in his estimate of the remedy employed in them. "The preceding facts," he says, "seem unequivocally to show that when given boldly and in large doses, the resin of hemp is capable of arresting effectually the progress of this formidable disease, and in a large proportion of cases of effecting a perfect cure;" and we are, moreover, after reviewing the whole of the evidence here submitted to us, willing to join with him in the belief, "that in hemp the profession has gained an anti-convulsive remedy of the greatest value."

We regard the profession as under great obligations to Dr. O'Shaughnessy for the publication of the important facts detailed in the pamphlet; and we hope this obligation will be not the less willingly admitted that the author shows as much modesty in preferring his claims to notice, as he exhibits philosophical caution in his experiments and in drawing conclusions from them.

